# The Unexpected Benefits of a Decolonized Knowledge Translation Initiative for Indigenous Mother Participants

**DOI:** 10.1177/10497323231167308

**Published:** 2023-04-14

**Authors:** Amy Wright, Rachel VanEvery, Heather Burnside, Kristena B. Lopez, Katie Kewageshig-Fyfe, Brenda Jacobs, Andrea E. M. Floyd, Era M. Ferron

**Affiliations:** 1 Lawrence S. Bloomberg Faculty of Nursing, 70379 University of Toronto, Toronto, ON, Canada; 2 Department of Health, Aging and Society, 539067 McMaster University, Hamilton, ON, Canada; 3 Hamilton Regional Indian Centre, Hamilton, ON, Canada; 4 Compass Community Health, Hamilton, ON, Canada

**Keywords:** Indigenous knowledge translation, knowledge translation, trauma- and violence-informed care

## Abstract

Western health research’s approach to knowledge translation (KT) has been criticized by Indigenous scholars, leaders, and communities for its misalignment with Indigenous ways of knowing and relational approaches to sharing knowledge. Conversely, Indigenous KT is understood as ‘sharing what we know about living a good life’ (Kaplan-Myrth & Smylie, 2006). Whereas KT in Euro-Western science contexts focuses on closing the ***know-do*** gap implying a separation of knowledge and action, knowledge in the Indigenous context is inherently practical and based on centuries old practices including oral traditions, experiential knowledge, and cross-cultural sharing. This article describes the development of a decolonized KT strategy. This community-engaged KT initiative was developed at the suggestion of Indigenous mothers who participated in a research study in Hamilton, Canada, which examined their experiences using health care to meet the health needs of their infants. Indigenous mothers participated in three main roles related to the KT activities: sharing their story in video, participating as team members on an Advisory Board, and directing the creation of a video series and website educational resource (KT strategy). Five Indigenous mothers participated as members of the Advisory Board. The process of participating had positive impacts on the mothers, namely, empowerment, strength, ‘I am not alone’, and healing. These unexpected findings, which go beyond the original project purpose to create an educational resource, show the significant and important benefits for research participants, particularly those from Indigenous communities, to be involved in decolonized KT strategies.

## Background

Knowledge translation (KT) has been described as ‘the process(es) through which knowledge is transformed into strategic action’ (Estey, 2008, p. 7). This definition is widely accepted and implies that knowledge is gained through a systematic, evidence-based approach with the goal of putting knowledge into practice that is meaningful for the end-user (Smylie et al., 2004). Despite this, however, the concept of KT has been criticized by Indigenous scholars, leaders, and communities. Western-based KT separates knowledge from action, whereas in the Indigenous context, KT is inherent and intrinsic (Morton Ninomiya et al., 2017; Smylie et al., 2004). Indigenous concepts of knowledge have been described as holistic, relational, timeless, infinite, communal, oral, and narrative-based (Morton Ninomiya et al., 2017; Smylie et al., 2004). Indigenous scholars argue that generalized KT activities reflect colonized practices as they assume what is ‘best’ for Indigenous Peoples. However, a decolonized approach rooted in self-determination facilitates Indigenous Peoples to be co-creators and leaders in the research and KT process, including applying knowledge to local contexts (Morton Ninomiya et al., 2017, 2022).

In this article, the authors describe an Indigenous-led approach to KT, the purpose of which was to share research findings in a directly applicable way to health providers to initiate and promote change that will benefit Indigenous families and communities. The project will be described in depth later, but briefly, for the purposes of situating the project within the literature, the original research project involved urban-dwelling Indigenous mothers with young infants and aimed to understand their experiences using health care to care for their children. During data collection, many participants noted their desire to share their experiences with health providers in order to promote culturally safe health care and improve health care interactions for themselves and others. A few participants thought creating a video to visually share their stories with health providers could be an effective way to initiate positive change. Following completion of the research study, funding to create the suggested videos was obtained, and an Advisory Board was struck to oversee the design and production of the videos. The Advisory Board consisted of mothers, who now participated as group leaders, other members of the local Indigenous community, health providers, and researchers. We had not anticipated that the *process* of creating the video series and associated educational website would be as meaningful to the mothers as the products themselves. The benefits described by the mothers below were unexpected by the mothers themselves as well as the researchers and other group members. Certainly, the products of the KT initiative were created for their presumed benefit for mothers and Indigenous families, but the process of creating the videos and the mothers’ participation on the Advisory Board in positions of leadership were unexpected prior to embarking on the project. This article describes the benefits realized by the mothers through their participation in leading this KT initiative as a way to inform and encourage other researchers to consider similar involvement of research participants in KT strategies.

Kaplan-Myrth and Smylie (2006) described Indigenous KT in health research as ‘Indigenously-led sharing of culturally relevant and useful health information and practices to improve Indigenous health status, policy, services and programs’ (p. 24–25) or more simply put, ‘sharing what we know about living a good life’ (p. 25). Knowledge generation and KT strategies are intrinsically linked, therefore, as it is the responsibility of researchers to ensure that new knowledge and understanding leads to action as directed and prioritized by the community from which the knowledge originates. Jackson and Masching’s (2016) has further described the essential principles of Indigenous KT. According to the authors, Indigenous KT is composed of five methodological features: (1) drawing on Indigenous ways of knowing (which are relational, ecological, holistic, experiential, communal, and oral-based in nature); (2) decolonizing in its approach (interrelated approaches such that research findings are returned back to Indigenous people in culturally appropriate ways and in a language that is understood); (3) supporting self-determination (accomplished by focusing on the social-cultural contexts of Indigenous people); (4) being grounded in participatory approaches (principles of co-learning, mutual benefit, long-term commitment, and a relationship between academic and community partners); and (5) enacting ethical or moral responsibility to share research findings through enacting an ‘ethical space’ (Ermine, 2007). Ethical space allows for dialogue between two separate knowledge systems, cultures, and world views, and emphasizes working together without hierarchies. Historically, Indigenous communities have been viewed and treated as ‘others’, whereas Euro-Western groups are considered the authoritative, knowing body (Sunseri, 2007). According to Sunseri (2007), Euro-Western research – ‘a major vehicle for representing Indigenous peoples as the o*ther*’ (p. 97) – has led Western society to attribute negative characteristics to Indigenous Peoples. For example, Indigenous women have been disproportionately impacted by government-imposed poverty due to a lack of funding to social services on reserves (National Inquiry into Missing and Murdered Indigenous Women and Girls, 2019). Poverty and negative stereotypes have led to child service workers often scrutinizing Indigenous women as unfit mothers (National Inquiry into Missing and Murdered Indigenous Women and Girls, 2019), as found in our work described in more detail below. This colonialist agenda towards Indigenous Peoples, while changing, has dominated the Western research world. Consequently, Indigenous voices are often missing or absent from research.

While Indigenous people are often marginalized in Western-based research, Indigenous participants who engage in research may find several benefits. Research shows that participating in qualitative data collection strategies can, at times, have several benefits for participants beyond the research objectives. While research interviews are not counselling sessions, participants’ experiences of sharing personal information with an observant listener can be therapeutic (Birch & Miller, 2000; Campbell et al., 2010; Carter et al., 2008; Clark, 2010; Shamai, 2003). The act of disclosing intimate details of one’s life, including traumatic or violent experiences, provides the interviewee with the opportunity to reflect and re-examine past events, which can lead to personal change (Birch & Miller, 2000). Some research participants have reported their interview experience to be enjoyable and cathartic, motivated by the belief that their participation in research can help others (Carter et al., 2008). Other reported therapeutic effects of engaging in interviews have included developing a sense of self-worth, self-determination, empowerment, and healing as an outcome of self-reflection (Birch & Miller, 2000; Campbell et al., 2010; Clark, 2010; Shamai, 2003).

Similar to participating in research, participating in KT initiatives can lead to several unintended benefits. The systematic review by Morton Ninomiya et al. (2022) of KT approaches and practices in Indigenous research serves as an illustration. Their review of the literature uncovered that participating in KT increases self-determination and self-governance in Indigenous primary health care and child and youth mental health service contexts.

Because of the historical trauma of colonization, that continues to negatively impact Indigenous Peoples in what is now known as Canada, we grounded our approach to KT in a trauma- and violence-informed care (TVIC) approach. TVIC was originally developed as an approach to health care, in which a clinician strives to create a safe space for clients or patients by understanding the effects of trauma on behaviour and health outcomes, recognizing the role of power relations, and adjusting their care accordingly. TVIC also considers the impacts of the broader context on a person’s life, including systemic and interpersonal violence and structural inequities, while at the same time, emphasizing the traumatic impacts of historical and ongoing violence. In this project, an Advisory Board was formed at the outset, consisting of First Nations and Inuit mothers, First Nations early childhood service providers, a First Nations Knowledge Holder, health providers, and First Nations and non-Indigenous researchers. The Advisory Board led the project, at the direction of its Indigenous members. The Advisory Board members had a foundational understanding of the trauma and violence Indigenous Peoples often experience, and everyone did their best to ensure a safe space for sharing, by enacting a non-judgemental and respectful demeanour, and holding meetings in a private environment. A First Nations Knowledge Holder was present throughout and opened each meeting with prayer. She intervened when individuals became distressed by sharing relevant teachings and engaging the group in smudging. This was effective in creating a safe space conducive to sharing traumatic experiences.

## Methods

This community-engaged KT initiative was developed at the suggestion of Indigenous mothers who participated in a research study which examined their experiences using health care services in Hamilton, Canada, to meet the health needs of their infants. Details about the original study and its methodology can be found elsewhere (Wright et al., 2019a; 2019b; 2019c). Briefly, self-identifying Indigenous mothers with infants under two years of age shared their experiences using health care services to meet the needs of their infants. Health care experiences ranged from primary care contexts to acute care (emergency department and in-patient hospital services) and health promotional programming (community-based and in some cases, Indigenous-led services and programs). Findings generally related to experiences of racism and discrimination, and feelings of being judged as parents and the need for culturally safe care that appropriately met their needs in relational, respectful, and strength-based ways. Many of the mothers expressed an interest to share their experiences with health providers to create meaningful practice change that would improve access to care and care experiences for other Indigenous families. Some felt this would be most impactful if shared through video. In response, and in collaboration with the local Indigenous Friendship Centre, the Hamilton Regional Indian Centre (HRIC), the first author sought funding to support the creation of knowledge dissemination tools, specifically, a video series with the aim of sharing the mothers’ important messages with health providers through an online educational website. The intent of the project was to share the mothers’ health care experiences with health providers to demonstrate the impact that health providers have on Indigenous mothers and families by the way they provide care, and to suggest ways that care in any context might be adjusted to be more culturally safe and promote more positive experiences for families. Funding was granted by the Canadian Institutes of Health Research (CIHR) through a knowledge dissemination grant, which financially enabled the production of the Healing the Hurt video series, and provided compensation to an Advisory Board, including a group of Indigenous mothers. The original study was approved by the Hamilton Integrated Research Ethics Board. The KT project itself did not require ethical approval as it was an extension of the original project as knowledge dissemination, and all participants acted as members of the Advisory Board leading and directing the project. All members of the Advisory Board have been offered authorship, and all but one mother accepted this responsibility. The final mother who did not wish to be an author has been thanked for her participation in the acknowledgement section of the article.

### Knowledge Dissemination Activities

#### Healing the Hurt Advisory Board

The following situates the authors within the context of the project and the original research project itself. First, the initial Advisory Board consisted of five members, including two First Nations staff members from the HRIC – a Knowledge Holder and family advocate, an Inuit mother, a First Nations mother and nurse who acted as a research assistant on the project, and two non-Indigenous health professionals (one was also the lead researcher and first author). Furthermore, the first author is a non-Indigenous researcher and nurse practitioner with European settler ancestry. She has worked with the Friendship Centre and local Indigenous community in a variety of community-led projects over the last 10 years and developed strong, respectful relationships with staff at the Friendship Centre and other Indigenous-led organizations, as well as members of the Indigenous community across Southern Ontario. The research projects she has engaged in with members of the Indigenous community are a result of community-led research priorities, and her aim is to facilitate meeting their goals at their direction. As such, the Advisory Board was created to oversee the KT project in its entirety and from its inception. After funding was obtained, the membership of the group expanded to include more Indigenous mothers and First Nations research assistants. Although the Advisory Board was facilitated by the lead researcher (first author) and a First Nations research assistant (second author), all questions and decisions were addressed by the group through consensus. The group was united in its purpose and did not have disagreements that could not be quickly addressed through discussion. The videos were created through in person meetings over a period of one year and completed just prior to the COVID-19 pandemic. The resulting educational website (https://www.indigenousmomandbaby.org/) was completed shortly after, and the social media campaign is ongoing through 2023.

#### Advisory Board Process

The Advisory Board met several times to determine the purpose of the video series, review and select a video production team, and create content maps of each proposed video. The members stressed the importance of the group meetings being in a familiar and accessible space for the mothers, so meetings took place at the HRIC. Meetings were also conducted in respectful ways, and each began with prayer, teaching, and smudging led by the Knowledge Holder. The group prioritized ensuring a safe space, where each member was heard.

Following agreement on the project aims, the group canvased local Indigenous mothers, including those from the initial study, for volunteers to share their experiences on video. Five mothers, including two from the original Advisory Board, were interested in the opportunity and met with the Advisory Board to share their stories. One mother decided not to share her story on camera, and another felt her story was not as powerful as the remaining three, and thus deferred to them. The video production team and Advisory Board agreed that three mothers had the most potential to convey the importance of equitable and culturally safe health care by sharing their experiences on camera. Nevertheless, all five of these mothers became members of the Advisory Board, which had a final membership of nine members (one First Nations Knowledge Holder, one First Nations early childhood service provider, two health providers, one of whom was the main researcher, a First Nations research assistant and mother, three other First Nations mothers, and one Inuit mother).

#### Creating the Video Series

The video production team consisted of two main members, a videographer and a production manager. Both members met with the Advisory Board and mothers several times before filming. This allowed for a relationship and rapport to be built between the mothers and the video team in a safe space with fellow mothers and the Advisory Board. The filming took place on a single day, with the lead researcher and research assistant present to offer support. Despite sharing emotional experiences on camera, all mothers voiced how important it was to share their stories to improve health care for other mothers. The lead researcher was in contact with all three mothers at several points after filming to ensure they were emotionally and mentally well. The video production team edited the film and presented two videos and a trailer to the Advisory Board for feedback. All group members approved the final videos and website.

#### Educational Website for Health Care Providers

The videos are housed on the website (https://www.indigenousmomandbaby.org/) along with educational resources and further detail of the Advisory Board members and the project. The website has been designed to augment Indigenous cultural safety curricula and continuing education for health professionals and students of health professional programs in Canada. Both the website and a communication plan for sharing the site with educational programs and health care organizations were developed in collaboration with the Advisory Board.

## Results

Three mothers participated in filming their experiences of accessing health care for their infants in Hamilton. These mothers were enthusiastic about sharing their personal reflections and experiences about the overall process of the video development. The mothers also felt it was particularly important to share with other researchers by way of publication, the importance of including research participants, like themselves, in knowledge dissemination activities such as this one. The following section describes four benefits the mothers shared of their experiences participating in the video project. These four benefits include *empowerment, strength, healing, and ‘I am not alone’.*

### Empowerment

The experience of being involved throughout the process of developing the video series and sharing their stories with health providers was empowering for mothers. This approach to knowledge dissemination, which came from the mothers who participated in the initial research study and in creating the videos, found this strategy meaningful for them. For all three mothers, this was the first time they had been asked about their experiences with health care, and they found that sharing their experiences was empowering. In particular, they felt that sharing their story visually gave them power, and that sharing their story through video was more likely to have a greater impact on health professionals than their story in a journal article. For example, one mother shared, ‘I think there’s a big difference when you read something on paper versus hearing and seeing someone on video…I think it puts a face to us, I am not just a story’.

Research findings are commonly shared in written documents and consumed by academics. The mothers recognized this and expressed the importance of their stories reaching a broader audience of health providers. The mothers’ participation as members of the Advisory Board, responsible for guiding the video creation process and providing feedback throughout, led to feelings of ownership and pride – mothers were proud of themselves for being involved in the project, for making their stories public, in the hopes that their stories will encourage health providers to provide culturally safe care to Indigenous mothers and families. For example, one mother shared: ‘Seeing and hearing myself like was like “wow”…I can see it and it was so empowering to get up there and speak the truth and know these doctors are going to see this’. These examples of empowerment demonstrate the positive impact that including research participants in knowledge dissemination activities can have, both on the participants themselves as well as the effectiveness of the knowledge dissemination tool. Putting the power and control back into the hands of the mothers was particularly important in this project as Indigenous communities have historically been subject to exploitive research practices (Morton Ninomiya et al., 2017) and Indigenous women’s voices often go unheard (Sunseri, 2007).

### Strength

The creation of the videos allowed for the opportunity to acknowledge and recognize the strengths and resiliency of the mothers as well as the strengths of other Indigenous Peoples. Watching themselves on video provided an opportunity for mothers to reflect on their own strength as they had newfound recognition of how they had overcome significant challenges in their lives. One mother shared that she, ‘was able to see how strong other moms were and it was amazing and inspiring to me to see that even through all of what our people were put through, we are still here today as strong women and strong moms for our families’. Mothers participated in Advisory Board meetings, which provided an opportunity to hear the experiences of others and to learn more about the strengths of their community. Recognizing their own strengths was at times difficult, because of a history of being put down or made to feel less-than in situations. The Advisory Board provided mothers with a sense of control and ownership over the project, where mothers felt a responsibility to ensure the videos represented them and their stories in the way they wanted to be perceived. This was done by continuously sharing their feedback and guidance throughout the video development process. Furthermore, the researcher, knowledge keeper, and health provider on the Advisory Board made it a priority to consistently acknowledge the strength and resiliency of the mothers and discussed this often to minimize power imbalances within the group and ensure the mothers had a leadership role in the process.

### ‘I Am Not Alone’

Participating in the video project provided mothers with the opportunity to share their stories with other mothers, which brought about the recognition that their experiences were not unique and that many other Indigenous mothers and families have been treated similarly. One mother shared: ‘I knew that I was not alone in my experiences but talking about this sort of thing isn’t something people do regularly. Although it makes me upset to meet these moms and hear what they went through, it was comforting to know that I was not the only one who has had a negative experience in health care’. The mothers also acknowledged that while these experiences are not uncommon, they are not often talked about. One mother repeatedly used the phrase ‘I am not alone’ to describe her experience as she is aware of and recognizes that Indigenous people often experience difficult and negative interactions with health care providers. Recognizing that others have undergone similar experiences brought about a sense of comradery and solidified a sense of purpose in the group to create a video product that conveyed the message to health providers that change in health care delivery is necessary.

### Healing

Arguably, the most significant theme throughout this project is the concept of healing. The mothers shared that their experience on the Advisory Board and creating these videos provided an opportunity to heal. Seeing and hearing themselves on video was therapeutic by encouraging them to ‘unpack’ and process their feelings. One mother shared that this experience had led her to seek professional counselling and shared, ‘It made me stop running–I couldn’t run from certain things anymore…It was needed, and I have to look at it as growing pains’. She acknowledged that while it was initially difficult to see and hear herself speak about her story on video, it led her to find help, which began her healing journey. Another mother shared how simply being invited to participate in the project and having space made for her to share within the group was an opportunity for healing. Sharing her experiences of trauma during her pregnancy and the loss of her infant with the Advisory Board and again on film changed her perspective on childbearing – ‘I felt like it allowed me to process what happened and before this experience, I thought there was no way I would ever have children again because I was so scared from what I went through the first time around. Now I feel like I want more children and I am excited for the future’. The mothers felt safe at the Advisory Board meetings where they could share their experiences, learn from one another, and be supported by each other. These meetings in themselves represented a type of therapy for mothers who often had not otherwise felt listened to or cared for. Finally, the opportunity to share their stories with the aim of improving care for future mothers and families was a way to change their pain and negative experiences into something good.

## Discussion

Our community-engaged KT strategy had several benefits for the participant mothers beyond the primary project aim to create an educational video series for health providers. The project took a trauma- and violence-informed care (TVIC) approach, understanding that interpersonal violence and structural inequities impact a person’s behaviours and health outcomes (Gender Trauma & Violence Knowledge Incubator & Equip Health Care, 2021). We also incorporated Jackson and Masching’s (2016) principles of Indigenous KT. First, we drew on Indigenous ways of knowing by co-leading the project with Indigenous mothers who sat on the Advisory Board to make meaningful decisions about how their stories were told in the videos. The board meetings were a safe space where board members could congregate and relate with one another and where the participants could share stories. Second, we used a decolonizing approach by facilitating the mothers’ decision-making power as members of the Advisory Board, responsible for creating the videos alongside the production team. In this way, the videos, although intended to be used as educational tools for Euro-Western health care providers, will resonate with the Indigenous community as they were created by Indigenous mothers. The third decolonizing strategy that we incorporated was our participatory and TVIC approaches from project inception to final product. The mother participants felt empowered and became increasingly self-determined through their involvement at the decision-making table and the video creation process. Fourth, we took a community-engaged, participatory approach, including members of the Indigenous community on the Advisory Board, and privileging their voice over those of non-Indigenous members. Relationships were formed (and continue to be nurtured) well beyond the grant funding timeline. We believe that the relationships that were built over several years between the main researcher and members of the Indigenous community through the project’s partner organization, the HRIC, led to a project environment of co-learning, mutual benefit, and a long-term relationship between our academic group and the Indigenous community in the Hamilton area. Finally, we strived to ensure we created an ethical space for all members of the Advisory Board, to engage in respectful dialogue to work together to create an educational resource to address discrimination in health care.

Participating in creating a video series to share their experiences with health providers was impactful for the mothers in several ways. The mothers shared that they felt empowered, came to recognize new strengths, and no longer felt alone, and that sharing their stories publicly promoted healing from past trauma. These are significant and important benefits for research participants, particularly those from Indigenous communities who are often marginalized by Western-based research. The mothers took the opportunity to share their experiences and through doing so found their voices amplified by creating impactful videos. As members of the Advisory Board, the mothers were in a position of power and authority, as they helped to direct the project by making decisions on the process, video team, and how their stories were shared. This was not just empowering but also helped illuminate their strengths, as they simultaneously recognized the strength of other mothers working with them as members of the Advisory Board. The mothers were validated throughout this process, not only by other members of the Advisory Board but also by the other mothers participating who had similar experiences. This led to a sense of commonality and belonging within the Advisory Board and prompted embarking on a healing journey for the mothers.

### Lessons Learned

Social justice work does not stop after the research ends. Researchers should strive to continue the work they start by bringing to light social injustices by continuing to augment the voices of research participants during KT activities. In particular, KT activities should be at the direction of the communities involved in the research in the first place. Often those involved in the research have impactful suggestions for sharing their stories and creating meaningful change that will impact their own lives (Morton Ninomiya et al., 2017). For researchers involved in social justice work, there is also an obligation to ensure that the research makes a direct and positive contribution to the lives of the participants [such as adhering to the 4Rs of research – respect, relevance, reciprocity, and responsibility (Kirkness & Barnhardt, 1991)]. Through engaging research participants in KT activities, the researcher puts the power back into the hands of the participants, having them guide the direction of dissemination strategies and ensuring outputs are approved by the participants themselves (Shamai, 2003). This is not to suggest that the ownness should be placed on the participants to also do the work of creating knowledge dissemination products, but instead, participants and the community should be involved and have the power to guide and direct the sharing of their data to ensure it is done appropriately, effectively, and in culturally safe ways.

Next, researchers must recognize the potential for re-traumatization when engaging research participants in disseminating research findings. Depending on the KT strategy, participants may be required to share their experiences multiple times beyond the initial interview. In the case of the video series, the mothers were asked to recount their stories in an Advisory Board meeting and again on video. These were emotionally triggering events for the mothers to recount. And while they reported after the fact that sharing their stories was actually healing for them, this was not known or realized immediately afterwards. In this situation, the lead researcher, also a nurse practitioner, felt compelled to follow up with the mothers several times over the next few months, to ensure they were emotionally and mentally well. Although the video dissemination activity was conceptualized and developed by the mothers, it was not pre-emptively realized that this would be potentially harmful for the mothers. This was an important lesson learned and one that is necessary to share with other researchers to ensure this potential harm is considered and protective strategies are put in place. Researchers should take care to have strategies in place to care for research participants with a history of exposure to trauma and violence who are participating in KT, just as they would during data collection. Some ideas for these strategies include debriefing with participants after meetings, keeping in regular contact with participants, providing participants with mental health support resources (e.g. phone numbers) that they can use to seek support as necessary, and linking Indigenous participants to cultural supports, including Elders and/or Knowledge Holders/keepers (BC Provincial Mental Health and Substance Use Planning Council, 2013; Jefferson et al., 2021; Nonomura et al., 2020).

By considering TVIC principles, researchers who conduct research with Indigenous populations can frame their approach around participants’ experiences of trauma and violence as residing not just in the psychological state but also as a result of social circumstances. In our work, we implemented TVIC principles throughout data collection and KT activities by providing a safe space for the mothers to share their stories in their own words and at their own pace during Advisory Board meetings and video production, and through follow-up phone calls and texts.

Working with populations who have experienced and continue to experience violence can result in vicarious trauma among researchers (Coles et al., 2014). Without protective mechanisms, researchers may experience physical symptoms and emotional distress, such as anger, guilt, shame, fear, crying, and feelings of depression (Coles et al., 2014). To protect against vicarious trauma and improve well-being, researchers should engage in coping strategies identified for work-related trauma when working with participants. Coping strategies include being prepared for engagement with participants (such as having pamphlets with lists of supports for both the participant and the researcher, being sensitive to violence-related topics); using formal and informal debriefing with colleagues, supervisors, friends, family members, or professional counsellors; taking breaks to limit exposure to traumatic material whether field notes, audio recording, data entry and analysis, or report writing; and self-care strategies whether physical, creative, social, or spiritual in nature.

## Conclusions

Community-engaged, participatory KT initiatives can be beneficial for participants. Being involved in decision-making about KT processes and products with an Indigenous KT approach and TVIC lens positively impacted the Indigenous mother participants beyond the objectives of the original research study. When hierarchical structures of the academic research system are broken down to enable participants' ownership in how their story is told, it fosters their empowerment, enables them to see their own strength, brings about a sense of healing, and shows them that they are not alone in their traumatic experience. Researchers must keep in mind that traumatized participants may be re-traumatized as a result of participating in research where they are asked to share their experiences. Therefore, this type of research work requires a framework and mindset that recognizes the broad context (e.g. historical, systemic, and social) within which trauma and violence occur and the psychological, physical, and behavioural impacts on participants. By adopting the TVIC approach, researchers will purposefully create a safe space for participants to share their stories. Equally important is that researchers recognize the impact of vicarious trauma on themselves and the importance of engaging in coping strategies for their own well-being.
